# Procedures for assessing psychological predictors of injuries in circus artists: a pilot prospective study

**DOI:** 10.1186/1471-2288-14-77

**Published:** 2014-06-11

**Authors:** Ian Shrier, John S Raglin, Emily B Levitan, Murray A Mittleman, Russell J Steele, Janette Powell

**Affiliations:** 1 Centre for Clinical Epidemiology, Lady Davis Institute for Medical Research, Jewish General Hospital, 3755 Ch. Côte Ste-Catherine, Montréal H3T 1E2, Canada; 2 Kinesiology Department, Indiana University, Indianapolis, Indiana, USA; 3 Department of Epidemiology, University of Alabama at Birmingham School of Public Health, Birmingham, Alabama, USA; 4 Cardiovascular Epidemiology Research Unit, Department of Medicine, Harvard Medical School, Harvard University, Boston, Massachusetts, USA; 5 Department of Mathematics and Statistics, McGill University, Montreal, Canada; 6 Performance Medicine “O”, Cirque du Soleil, Las Vegas, USA

**Keywords:** Epidemiology, Injury, Psychological risk factors, Prospective

## Abstract

**Background:**

Research on psychological risk factors for injury has focused on stable traits. Our objective was to test the feasibility of a prospective longitudinal study designed to examine labile psychological states as risk factors of injury.

**Methods:**

We measured psychological traits at baseline (mood, ways of coping and anxiety), and psychological states every day (1-item questions on anxiety, sleep, fatigue, soreness, self-confidence) before performances in Cirque du Soleil artists of the show “O”. Additional questions were added once per week to better assess anxiety (20-item) and mood. Questionnaires were provided in English, French, Russian and Japanese. Injury and exposure data were extracted from electronic records that are kept as part of routine business practices.

**Results:**

The 43.9% (36/82) recruitment rate was more than expected. Most artists completed the baseline questionnaires in 15 min, a weekly questionnaire in <2 min and a daily questionnaire in <1 min. We improved the formatting of some questions during the study, and adapted the wording of other questions to improve clarity. There were no dropouts during the entire study, suggesting the questionnaires were appropriate in content and length. Results for sample size calculations depend on the number of artists followed and the minimal important difference in injury rates, but in general, preclude a purely prospective study with daily data collection because of the long follow-up required. However, a prospective nested case-crossover design with data collection bi-weekly and at the time of injury appears feasible.

**Conclusion:**

A prospective study collecting psychological state data from subjects who train and work regularly together is feasible, but sample size calculations suggest that the optimal study design would use prospective nested case-crossover methodology.

## Background

Participation in physical activities is associated with an increase in quality of life [1], enhanced academic performance [2-4], a positive impact on behavior [5-7], better mental health [8], better physical health [9,10], and decreased obesity [11]. Despite its benefits, an increase in physical activity is also associated with an increased injury risk. An estimated 20.6 million children are injured each year in the USA [12], and 559,000 in Canada [13]. Of those receiving medical attention for sports and recreation related injuries in the US, one fifth of schoolchildren and more than one quarter of working adults experience one or more days of lost time from school or work [14]. In addition to these short-term effects, injured athletes may have an increased risk of long-term sequelae such as osteoarthritis with concomitant reduction of physical capacity [15-17]. A better understanding of the causes of sport injuries and their recurrences can therefore have a significant impact on athlete health and associated care costs.

In addition to physical, equipment, or procedural causes of activity-related injuries, psychological risk factors may play an important role. Although early psychological research was often limited to theoretical tests of single variables (e.g., personality) more recent work has utilized a multi-variable stress and sport injury model [18]. Some findings indicate the experience of stressful life events can increase injury rates [18,19], although its impact varies across sports [20] and is often moderated by social support and coping styles [20,21]. Subsequently, other researchers amended the theory and utilized a variety of different scales to assess life events and coping, focusing on either sport or athletes [22].

Despite this work, a literature review found only “limited scientific knowledge” [20] while suggesting that situational-dependent emotional states can also influence injury. Although high anxiety may worsen performance and increase the risk of injury, its impact is quite variable among athletes, even among those with similar skills competing in the same event [23,24]. Studies based on Hanin’s Individual Zones of Optimal Functioning (IZOF) [23] have found that 25-45% of athletes actually require high anxiety to achieve good or optimal performances, yet to date no prospective research has examined sport injury using this perspective (one study used a retrospective design [25]).

Unpleasant moods are also associated with increased injury risk [20,26]. However, these authors utilized a single baseline assessment of mood, often with a considerable lag time between psychological assessment and injury. Assessment of mood at regular intervals would be far more efficacious given the dynamic nature of athletic training load, mood disturbance [27], and overtraining syndrome [27,28]. Depression, irrespective of whether it is the consequence of a negative life event (e.g., divorce) or is sport related (e.g., overtraining), would be expected to compromise the ability to concentrate and focus [29], and therefore increase injury risk [30].

Other psychological variables associated with injury include bodily self-perceptions (e.g., fatigue) [31], coping skills and life events [32], again assessed with only limited longitudinal research. Fawkner and colleagues [33] assessed daily hassles in 98 athletes weekly over a 13-week season. Injured athletes reported increased hassles in the week prior to injury compared with uninjured athletes, but the analysis grouped participants by outcome and then observed exposures without considering that both exposure and covariates varied over time, a known source of potential bias [34,35]. Ivarsson and colleagues conducted two studies that included assessing whether the Daily Hassles Questionnaire predicted injury in Swedish Division 4–6 (competitive) and Premier League (professional) soccer players who completed the questionnaire weekly over a three-month period [36,37]. Unfortunately biases may have been introduced because the analyses could not account for time-varying nature of exposure or other covariates, and did not assess any state constructs aside from the Daily Hassles Questionnaire. In a third study on elite junior soccer players [38], the same group found differences in a trajectory analysis comparing Daily Hassles and Uplifting events among injured and non-injured subjects.

These initial studies have been informative, but there are important limitations to the methods that were used to address the role of psychological states that are believed to change frequently over time when the outcome is injury. These are explained below in more depth but in brief, analyses that compare scores for injured versus non-injured subjects address the question whether psychological factors differ between these two groups, rather than whether the varying psychological states within an individual affect injury risk. Because psychological states will correlate with psychological traits (and psychological traits are risk factors for injury), the above analyses did not address the question we are posing. If the psychological state is truly a risk factor within an individual, then psychological interventions on the day of competition would be promising to explore. Otherwise, one should focus on longer-term interventions that address the more stable psychological traits that have already been identified as risk factors (although differentiating causal from non-causal risk factors remains to be determined).

### Injury research strategies to minimize bias

When time-varying exposures and covariates are considered important, one could use two general approaches to minimize bias. First, one could ask patients to recall how they felt before the injury. The control group could consist of non-injured participants at the time of injury (case–control study, where control participants are sampled at the time a case is diagnosed, and therefore can later be considered cases), or the same participants recalling their “states” a few days earlier (i.e., case-crossover study) [35]. However, these study designs are subject to potential recall biases.

Alternatively in a prospective design one measures psychological variables regularly (e.g. daily or weekly), where the analytical approach could be a longitudinal repeated-measures design (e.g. time-series) or a nested case-crossover analysis. In this approach sample size requirements are dependent on both the frequency of injury events and the correlation of psychological states over time (higher sample sizes if states are relatively stable because there is less information on the changing exposures). However, this option involves logistical challenges associated with frequent collection of psychological data. Some psychological questionnaires may evoke scepticism among athletes, staff and coaches [39], and there may be variations due to translation [40], need to minimize time commitment [40,41], sport context, and participants’ understanding for the need to avoid response distortion (either faking good or bad) [42]. Completing daily questionnaires may result in fatigue, with answers provided in an unvarying or random manner [43], or drop-out. There are also challenges in managing daily data collection, and transmitting it to a central data management unit in any sport setting. Therefore, it is paramount to establish procedures and practices that facilitate these processes.

As a consequence, the primary objective of this pilot study was to test the feasibility of a prospective study specifically designed to test if psychological states are predictive of injury. More specifically, our objectives were to determine 1) time required to complete psychological questionnaires distributed at baseline (assessing traits), daily (assessing states), and once per week (assessing states including an additional questionnaire to assess mood); 2) optimal distributing and collecting of questionnaires to be completed daily or once per week; 3) questionnaire comprehension and acceptability and; 4) intra-individual correlation for daily psychological states necessary for sample size calculations in a larger longitudinal repeated-measures investigation.

The study population were artists from the show “O” produced by Cirque du Soleil (CDS). These artists come from a variety of athletic backgrounds including swimming, diving, gymnastics and acrosport, as well as performing arts including dancers and musicians. We chose this population because injury records and exposures are stored electronically, and conducting the study appeared feasible. Overall injury rates have been estimated to be 9.7 injuries per 1000 artist-exposures (similar to Men’s NCAA Basketball [44]), with 4.4% of injuries resulting in missing 15 or more performances (approximately equivalent to 10 days) [45]. We therefore expect these results to be useful in other athletic contexts involving regular psychological assessments for an extended period.

## Methods

### Preliminary study development

The McGill University Institutional Review Board approved this pilot study. The questionnaires could only cover a limited number of constructs because participants were required to complete them daily. To determine which constructs we should investigate in our context (see Table 1), we used an iterative process. We first individually interviewed CDS personnel that were familiar with circus-related injuries: Director of Performance Medicine responsible for shows that change location, Director of Performance Medicine responsible for shows that don’t change location, the head coach, a choreographer, a supervising physiotherapist for one show, and the Senior Performance Psychologist. Following these meetings, we outlined areas of interest, their potential mechanisms of action, and identified corresponding psychological constructs. These variables and constructs were later verified with the same CDS personnel.

**Table 1:** Constructs and associated questionnaires used to measure traits and states

**Construct**	**Questionnaire**	**Timing**
***Traits***		
Anxiety	STAI-Y2 [46]: A validated 20-item questionnaire.	Baseline
	STAI-Y1 Best [40]: A validated 20-item questionnaire measuring state anxiety relative to the time the artist had their best performance.	
Ability to cope, Frustration	Ways of Coping [47]: A validated 66-item questionnaire to identify the thoughts and acts used to cope in a specific stressful event. There are eight sub-scales in the student-sample scoring we used (problem focused, wishful thinking, detachment, seek social support, focusing on the positive, self blame, tension reduction and keeping to oneself). We chose the student sample scoring system because we felt that the problems associated with a production company environment (e.g. changeover of personnel, deadlines, new training) are more closely related to what students experience compared to a sample of stable community workers.	Baseline
Complainers, Unhappiness	Profile of Mood States (POMS) [48]: A validated 65-item questionnaire that includes subscales for tension, depression, anger, vigour, fatigue and confusion. There is considerable evidence that this global measure of mood disturbance is closely associated with the stress of athletic training [49] as well as relative success in athletics and other realms of performance with a significant physical component [30].	Baseline
***States***		
Conflicts, Anger, Frustration	Training Distress Scale [41]: A validated 7-item 5-point Likert scale questionnaire assessing specific and general mood states. Items generally come from the Profile of Mood States [48] Other items related to the construct of anger/frustration (identified by CDS management identified as potentially important) include “bad-tempered” and “peeved”.	Weekly
Anxiety	STAI-Y1 [50]: A validated 20-item questionnaire measuring state anxiety.	Weekly
Anxiety	Single-item Likert question [51].	Daily
Self-Confidence	Single-item 7-point Likert question [52]: This question examines an artist’s confidence about their upcoming performances that day. Variations of this single item are considered valid alternatives to more time consuming assessments of self-confidence [53], and are associated with anxiety and performance [52].	Daily
Poor sleep	Single-item Likert question [49].	Daily
Physical capacity	Fatigue: We used a 1-item validated question [54].	Daily
	General well-being: We used a 7-item Likert scale question (from Very very good to Very very bad) [49].	
	Sleep: 1-item questionnaire about the amount and quality of sleep [49].	
	Sickness within the last 24 hours.	
	Muscle soreness: 7-item Likert scale questions from Very Very Good to Very Very Sore [42,49] for whole body, legs and arms. The responses to these items have been found to be correlated to both more comprehensive psychological questionnaires [49], biological markers of stress such as cortisol [42] and objective measures of athletic performance [55].	

Table 1 illustrates the constructs identified as most likely to be important through the interviews with CDS personnel and a review of the literature, questionnaires chosen from the literature where they existed, and timing/frequency for questionnaire distribution. The constructs included trait and state anxiety, trait ability to cope, trait and state mood, and state confidence, where traits were measured at baseline and states were measured daily. We also included questions related to several state behavioural habits believed to influence injury risk. Our objective was to have artists complete the baseline questionnaires within 15 min, weekly questionnaires within 2 min and daily questionnaires within 1 min. Details of the specific questionnaires are described below.

### Recruitment

Approximately one month prior to the study, we approached all CDS artists ≥ 18 years of age working at the show “O” located in Las Vegas, Nevada. During a regular weekly meeting, we gave a brief 10-min presentation explaining the general principles of the study, and distributed consent forms. We emphasized that we could prepare reports for each individual showing how their daily profiles changed over time. In addition, because artists often rely on each other (e.g. catcher and flyer in a trapeze act), we emphasized that the information may be important to the team, even if the individual did not feel it was personally important (which would be analogous in some team sport contexts). Artists were asked to submit signed consent forms within one week if they were interested in participating.

### Questionnaire timing and processes

We provided the questionnaires in the four principal languages of the artists: English, French, Japanese and Russian. We used existing translations where available, and used the professional CDS translation service when necessary. At least one artist with conversational English language skills and whose first language was the translated language reviewed all translated questionnaires. Although we recognize that cultural differences exist between populations using these languages that might increase the heterogeneity of responses between participants, this should not affect our ability to reach our primary objectives of determining feasibility, and variance estimates for sample size calculations of a definitive study.

After discussions with the stage manager and artistic directors, baseline questionnaires including State-Trait Anxiety Inventory (STAI) [50], Ways of Coping [40,47] and Profile of Mood States (POMS) [48] were distributed at the time the artist provided informed consent, and were completed at home over the ensuing 2–4 weeks. On each working day, we placed a short “states” questionnaire (see Additional file 1) that included either 7 or 9 items (i.e. 2 additional questions included on the “weekly” questionnaire) at each artist’s make-up station (each had their own station). The items included in these questionnaires came from validated questionnaires where possible and included the constructs conflicts, anxiety, self-confidence, sleep and physical capacity (Table 1). Artists completed the questionnaire prior to the day’s performances, and dropped them into a locked drop-box or handed them to the supervising therapist. To minimize missing data, distributed questionnaires included the date and the artist’s identification number. Completed forms were scanned and emailed to the primary investigator for data entry.

Artists were encouraged to provide feedback throughout the study, with special reference to the time required to complete questionnaires, undesirable questions, logistical problems, workload, and possible duration for a definitive study.

### Injury and exposure data

To track injury and exposure data, we used the same methods as our previous studies [45]. In brief, CDS therapists use electronic charting for all injuries and treatments (provided free of charge to artists) through in-house injury tracking software. We classified injuries according to commonly used criteria in the sport injury literature [56-58] and we have used in the past with the same company [45] and are common in sport medicine epidemiology: Medical Attention (injury reported to a show therapist) and Time-Loss-1 (TL-1) if the injury resulted in at least one missed performance. Anytime an artist misses a performance, it must be documented as either a work-related injury (included in our analyses and for which the artist is covered by health insurance), or a personal health condition (excluded from our analyses). Exposure data were obtained from in-house software that keeps track of when artists are performing.

### Analyses

As a pilot study designed primarily to assess feasibility, we provide descriptive statistics only. For continuous variables, we report the median and interquartile region (IQR) because the data were skewed, and we report percent for categorical variables. In addition, we report the direct feedback elicited during weekly meetings. Our sample size was determined by the number of artists within the show and was not modifiable. For practical considerations due to vacation time, we used an 8-week period that began one week after a vacation and ended within two weeks of the next vacation.

To calculate sample sizes for the definitive study, we used linear mixed models and generalized estimating equations (GEE) to account for the intra-participant correlations for the psychological variables over days (stability of states across time), and took a precision-based approach for the estimate via simulations based on psychological daily data that corresponded to our observed data. The observed injury rates (shown in results) were much less than previous studies in the general CDS population. Given the short time frame of the pilot study, we felt the results from our previous study would provide a more reliable estimate. We estimated a baseline TL-1 injury rate of 1.6 injuries per 1000 artist-exposures for the company [45], and a minimal important difference (MID) of 25% (i.e. 1.2 injuries per 1000 artist-exposures). We calculated sample sizes based on at least a 2-category change in an ordinal scale (all daily questions used essentially ordinal data). We varied the number of artists (between 180–1000) and the number of observation days (40–250) to explore different assumptions (always setting alpha to 0.05, 2-sided). All simulations and calculations were conducted in the open-software R [59] with the lme4 [60] and geepack [61] libraries. In addition, we estimated sample size for a case-crossover study using a matched case–control design using free open source statistical software [62] and the following parameter values: 2-sided alpha = 0.05, power = 0.8, 10:1 ratio of controls to cases, risk of exposure (categorized dichotomously) in controls at 0.3, and an MID of 25% and 50% increased risk of exposure in cases.

## Results

Of the 82 artists approached, 42 initially expressed interest. However, three declined after receiving the baseline questionnaires, and two took personal leave of absences before the study began, leaving 16 females and 21 males recruited for the study (45.1% recruitment). Artists’ region of origin included Canada, United States, Australia, Europe, Russia, and Asia. One of our four translations represented the first language of the artist in 31/37 artists, second language in 5/37 artists, and third language in 1/37. The median age was 32.4 (IQR: 29.2 to 37.9). We received feedback evaluations from 31/37 participating artists. The mean values for the baseline psychological questionnaires are provided in Table 2 for the 33 participants where there were no missing answers.

**Table 2:** Mean (sd) for each of the psychological questionnaires administered at baseline*

**Questionnaire**	**Subscale**	**Mean (sd)**
STAI-Y1		37.5 (12.4)
STAI-Y1 (Best)		31.6 (10.2)
STAI-Y2		38.8 (10.8)
POMS	Tension-Anxiety	14.7 (6.6)
	Depression-Dejection	7.9 (9.1)
	Anger-Hostility	8.8 (7.9)
	Vigor	20.0 (5.4)
	Fatigue	9.1 (5.7)
	Confusion-Bewilderment	12.2 (4.4)
Ways of coping	Problem focused	20.2 (5.2)
	Wishful thinking	7.1 (3.7)
	Detachment	8.3 (3.8)
	Seek social support	10.9 (4.7)
	Focusing on the positive	7.9 (2.6)
	Self blame	4.7 (2.1)
	Tension reduction	3.6 (1.5)
	Keep to self	3.9 (1.8)

*Values represent means for participants who had no missing values: n = 34 except for STAI-Y2 where n = 32.

### Distributing and collecting questionnaires

To facilitate the process, identification numbers on questionnaires were numbered in the same order as the participating artists’ make-up tables, but were not sequential (i.e. two proximal artists might be 63 and 66) in order to enhance confidentiality. Distributing the questionnaires required ~10 min and was usually done ~2 hours before the night’s first performance. In general, feedback from the artists suggested the ability to simply drop completed questionnaires in a locked box located near the door was well received.

### Time to complete questionnaires

The baseline questionnaire was completed in the targeted time (<15 min) by more than 60% of artists but required more than 30 min by two artists. We expected the weekly questionnaire to be completed in <2 min and the daily questionnaire to be completed in <1 min, but ~20% of artists required more time. That said, 45-50% of the artists required less than half the expected time. When we cross-correlated the results from the different questionnaires, the two participants requiring >30 min for the baseline questionnaire were not the same participants requiring longer periods for the weekly or daily questionnaires. Of the seven participants requiring >2 min for the weekly questionnaire, the time required for the daily questionnaire was <30 s for two artists, between 31–120 s for three artists, and >2 min for two artists.

### Acceptability and comprehension of the questionnaires

There were no questions that artists felt should not be asked. No artist felt the workload onerous, but a couple of artists felt it was bothersome to answer the same questions daily. Of the 26 participants who answered how much longer they would be willing to continue completing the questionnaires if the study was repeated, five responded as long as needed, five responded ~2-3 months, two responded one month, two simply said they would continue for now, three were unsure and nine said they would no longer continue.

Based on artist feedback, we made important changes to the text and format of the daily questionnaire within the first couple of weeks (see Additional file 1). The original versions of questions 5 and 6 were re-worded to clearly indicate they referred to the upcoming performance not yet done. We also added “current role” to question 6 to clearly indicate we were interested in the optimal level of anxiety relevant to their current role at CDS.

Despite our encouragement for feedback, the first question of the daily questionnaire (overall well-being, “How do you feel today?” with 7-point Likert response from Very very good to Very very bad) was left unanswered on 68/1297 collected questionnaires; several artists provided feedback saying it was difficult to give an “overall” assessment. The question regarding sleeping was left unanswered 42/1297 times, likely due to questionnaire formatting because we did not receive any comments. We believe adding a dotted line from the question to the answer choices might draw the artists’ eyes to the question-answer group.

### Injuries during pilot study

Over 2660 artist-performances, there were a total of nine Medical Attention injuries (3.4 injuries per 1000 artist-performances) and three TL-1 injuries 1.1 TL-1 injuries per 1000 artist-performances.

### Sample size calculations

Intra-participant correlations were higher than expected, and three artists had no variability within the study for some psychological factors. With the low TL-1 injury rate and low variability, sample size calculations for both the linear mixed and GEE models were very high, with the linear mixed model predictably showing slightly less power than the GEE results. For example, using 180 artists measured over 100 days would provide only 13% power under a GEE model. Increasing the number of artists to 1000 only increases power to 30%. Increasing the number of days of observation increases power as well, but following 750 artists for 250 days yields only 30% power, and following 1000 artists for 250 days yields only 61% power.

Based on these results, we consider it infeasible to reliably follow participants daily for so long. Therefore, we also calculated the sample size for a case-crossover study where we would measure artists only once or twice per week, and then again post-injury. Based on the parameters discussed in the methods, and the estimated intra-participant correlations ranging from 0.16 to 0.58 for the different variables measured (Table 3), we would need between 828 and 1301 cases (injuries) if the MID is 25% increased risk. The number of artist-performances needed to occur is equal to the required number of cases divided by the injury rate per 1000 artist exposures. With an expected TL-1 injury rate of ~1.5 per 1000 performances one would need to follow between 550,000 (for “ill past 24 hours”) to 870,000 artist-performances (for “confidence”). With recruitment similar to our pilot study of 35 artists per show on six shows (210 artists) and assuming an average of 470 performances per year, we would need to follow the artists between 67–105 months. If the MID is 50% increased risk, the number of cases and artist-exposures is greatly decreased (to ~30% of the above values), but we would still need to follow artists for 20–32 months.

**Table 3:** **Estimated Intra-class correlations (ICC)**^**1 **^**and calculated sample sizes**^**2 **^**for variables measured daily over the entire study**

**Variable**	**Estimated ICC**^**1**^	**Estimated sample size**^**2**^
Feeling today	0.35	950
Hours slept	0.34	941
Ill past 24 hours	0.16	828
Whole body soreness	0.41	1011
Art soreness	0.46	1075
Leg soreness	0.40	1000
Confidence	0.58	1301
Anxiety	0.56	1254
Fatigue	0.32	924

^1^ICC was calculated as within subject variance/total variance within a linear regression random effects model.

^2^Sample sizes calculated using matched case–control design and the following parameter values: alpha = 0.05, power = 0.8, 10:1 ratio of controls to cases, risk of exposure (categorized dichotomously) in controls at 0.3, and an increased risk of exposure in cases at 20%.

If instead one were interested in medical attention injuries, the estimated injury rate from previous studies is ~10 per 1000 performances. In this context, we would need to follow 210 artists for ~10-16 months (82,800 to 130,1000 artist performances) for an increased risk of 25%, but only ~ 3–5 months (24,200 to 38,900 artist performances) for an increased risk of 50%. Increasing the number of artists through improved recruitment per show, or by including additional shows would further decrease the amount of time required to complete the study.

## Discussion

This study examined procedures and methods to be used in a larger prospective study investigating the role of psychological states on injury. It required participants to complete psychological questionnaires of varying length on a daily basis and the 45% recruitment yield was higher than anticipated. There were also no dropouts among the artists who agreed to participate, indicating they found the workload and questionnaires to be generally acceptable, with the noted exception of providing an overall assessment of well-being.

### Psychological protocols

To maximize acceptability and participation by the artists and their support staff, we used a participatory research approach [63]. We sought input from the stakeholder’s key personnel in both the identification of the theoretical model and selection of psychological measures. We used an iterative process to ensure we captured all the salient features, and to prioritize the traits and states of most interest. To minimize dropouts, we targeted specific questionnaire completion times based on our previous experience in other similar studies, and on the opinions of the stakeholder. During the study, we altered the formatting and wording of some questions based on participants’ feedback.

In general, the majority of artists completed the questionnaires within the target time and we obtained data on state anxiety, mood, self-confidence, and physical factors that will be presented in other reports. From informal conversations, some artists were more sensitive to nuances within their own psychological states and required more time to distinguish among the various choices in the questionnaires. For interval validity, the artist should be consistent in her/his approach throughout the course of the study. We would expect the acceptable time to complete questionnaires in other activities or sports to be generally similar, but there may be important differences based on the participating group’s culture, and research requirements. Therefore, we encourage investigators using protocols that require repeated psychological assessments to engage stakeholders to build support and determine acceptable questionnaire lengths, and other important insights. We chose to prioritize our data on the assessment of labile states, moods and feelings rather than on distal events (e.g. conflict with management, which is a cause of mood). We did not include self-efficacy [64,65] because the analyses are expected to be confounded by ability, and therefore any interpretation would be questionable even if it also affects injury risk itself. Nor could we explore an artist’s body awareness (common in dancers), and nutrition habits. Although we have data on sleep patterns, CDS artists work at night and some regularly (or irregularly) take naps; these interim periods of sleep could theoretically reduce the effect of a poor night’s sleep.

### Sample size calculation

Sample size requirements for a definitive prospective study on time loss injuries is prohibitively large, but using an MID of 50% increased risk, one could use the case-crossover design with prospectively collected control data to investigate the role of psychological states by following 210 artists for ~2-2.5 years for TL-1 injuries, and for ~3-5 months for medical attention injuries. These large sample size requirements (both in number of participants, and duration of study which could result in subject fatigue) represent an important challenge for any study investigating the role of psychological states and injury. Sample size requirements would be reduced if the injury rate is higher than 10 injuries per 1000 exposures, the MID is higher than 50%, or the correlation of states over time is lower than in our study.

Our very large sample size calculations appear to conflict with previous prospective studies showing statistically significant results for psychological factors as risk factors. However, as previously mentioned, there are important differences between the studies. First, the TL-1 injury rate in previous data from our artists was 1.5 injuries per 1000 artist-performances. The injury rates estimated from the various published studies (assuming 6 exposures per week per athlete) were approximately 5.1 injuries per 1000 athlete-exposures (games or practices) [37], 6.9 injuries per 1000 athlete-exposures [36], 11.1 injuries per 1000 athlete-exposures [38], and 4.9 injuries per 1000 athlete-exposures [33]. The much higher injury rate in elite soccer compared to circus artists in our data suggests sample size calculations would be closer to our calculated Medical Attention injury definition. Second, our analyses address the question whether certain psychological states are risk factors for injury *among participants who get injured* (it is not possible to measure the effect in those that never get injured). Other published studies on psychological states [33,36-38] addressed whether psychological factors distinguish *between athletes who get injured and athletes who do not get injured*. The distinction is important because if the psychological state is important in those that get injured, interventions for the day of competition or training are promising for injury prevention. However, if the psychological state is not important in those that get injured and is just a marker for a psychological trait, then longer-term interventions must be designed to change the more stable psychological traits (if these are indeed causal factors rather than markers for true causal risk factors). Third, the previous studies compared injured to non-injured subjects, which is a case–control analysis. Because they did not use incidence-density sampling, the results may overestimate the effect if adverse psychological states decrease during the study. Finally, previous studies have focused on the Daily Hassles questionnaire (measured weekly) whereas we focused on daily states related to confidence, anxiety and mood. Ivarsson [38] estimated the ICC for the Daily Hassles questionnaire to be 73% in elite junior soccer players, which is higher than the ICC for the variables we report in Table 3. This suggests the required sample size would be higher rather than lower for questions addressing whether the psychological state affects injury risk in those that get injured.

Although retrospective case-crossover design is possible, the risk of recall bias is high. Another alternative is to use a hybrid approach where one prospectively collects “control” exposure data on psychological states only once or twice per week, and contrast this with psychological state data collected for the “case-event” after the injury occurred. In this study design, some of the cases would happen to occur on days where pre-performance exposure data had already been collected. Thus, one could estimate the magnitude of “case” recall bias by comparing exposures collected retrospectively after the injury with exposure data collected prospectively before the performance. There is research demonstrating athletes can provide accurate retrospective reports on past performances, at least for anxiety and pre-performance moods [40].

Finally, recent technological advances allow data to be entered directly via smartphones and tablets. Although these methods may appear promising, surveys on electronic devices are sometimes read/interpreted differently from paper-based systems, and response rates may or may not be improved [66-68]. Investigators should evaluate the advantages and disadvantages of all methods within the context of their study.

## Conclusion

We found that that the procedures used to implement systematic psychological monitoring of CDS artists were successful with respect to time to complete, distribution, acceptability and comprehension of questionnaires. The same methods could be implemented in other sport contexts where injury and exposures are closely monitored, and participants have their personal reserved space (e.g. in a dressing room) to receive and complete questionnaires. Challenges for recruitment and retention were primarily related to the degree of willingness to repeatedly complete questionnaires. Additional challenges include optimal questionnaire formatting, prioritizing which psychological constructs to study, and the limited number of questions due to time constraints. Finally, in the population studied, sample size calculations suggest several years of follow-up would be necessary to measure states that change daily given the injury rates. Sample sizes would be lower if injury rates were higher, or correlations between daily states lower.

## Competing interests

This study did not receive any funding. Ian Shrier is funded by the Lady Davis Institute for Medical Research, Jewish General Hospital. Ian Shrier is Consulting Medical Director for Cirque du Soleil. He did not receive any funding or payment for work related to this study. Janette Powell is a supervising physiotherapist at the Cirque du Soleil show “O”. No other authors have any other professional relationships.

## Authors’ contributions

IS conceived the study, and was involved with data collection, analysis and writing the manuscript. JSR was involved in the study design and writing the manuscript. EBL and MAM helped conceive the study, guide the analyses, and participated in writing the manuscript. RJS was involved in the study design, supervised the analyses and participated in writing the manuscript. JP was involved in the study design, data collection and in writing the manuscript. All authors read and approved the final manuscript.

## Pre-publication history

The pre-publication history for this paper can be accessed here:

http://www.biomedcentral.com/1471-2288/14/77/prepub

## Supplementary Material

Additional file 1 Daily questionnaire. Click here for file

## References

[B1] Ekstrand J Gillquist J Soccer injuries and their mechanisms: a prospective study Med Sci Sports Exerc 1983 15 267 270 10.1249/00005768-198315030-00014 6621313

[B2] Dwyer T Sallis JF Blizzard L Lazarus R Dean K Relation of academic performance to physical activity and fitness in children Pediatr Exerc Sci 2001 13 3 225 237

[B3] Sallis JF McKenzie TL Kolody B Lewis M Marshall S Rosengard P Effects of health-related physical education on academic achievement: project SPARK Res Q Exerc Sport 1999 70 2 127 134 10.1080/02701367.1999.10608030 10380244

[B4] Tremblay MS Inman JW Willms JD The relationship between physical activity, self-esteem and academic achievement in 12-year-old children Pediatr Exerc Sci 2000 12 3 312 323

[B5] Escobedo LG Marcus SE Holtzman D Giovino GA Sports participation, age at smoking initiation, and the risk of smoking among US high school students JAMA 1993 269 11 1391 1395 10.1001/jama.1993.03500110059035 8441214

[B6] Kulig K Brener ND McManus T Sexual activity and substance use among adolescents by category of physical activity plus team sports participation Arch Pediatr Adolesc Med 2003 157 9 905 912 10.1001/archpedi.157.9.905 12963597

[B7] Pate RR Trost SG Levin S Dowda M Sports participation and health-related behaviors among US youth Arch Pediatr Adolesc Med 2000 154 9 904 911 10.1001/archpedi.154.9.904 10980794

[B8] Kirkcaldy BD Shephard RJ Siefen RG The relationship between physical activity and self-image and problem behaviour among adolescents Soc Psychiatry Psychiatr Epidemiol 2002 37 11 544 550 10.1007/s00127-002-0554-7 12395145

[B9] Janz K Physical activity and bone development during childhood and adolescence. Implications for the prevention of osteoporosis Minerva Pediatr 2002 54 2 93 104 11981524

[B10] Janz KF Dawson JD Mahoney LT Increases in physical fitness during childhood improve cardiovascular health during adolescence: the Muscatine Study Int J Sports Med 2002 23 Suppl 1 S15 S21 12012257 10.1055/s-2002-28456

[B11] Hill JO Leon AS Leon AS Physical Activity, Body Weight, and Body fat Distribution Physical Activity and Cardiovascular Health A National Consensus 1997 Champaign, Il: Human Kinetics 88 97

[B12] Danseco ER Miller TR Spicer RS Incidence and costs of 1987–1994 childhood injuries: demographic breakdowns Pediatrics 2000 105 2 E27 10.1542/peds.105.2.e27 10654987

[B13] Pless IB Millar W Publications HC Unintentional Injuries in Childhood: Results from Canadian Health Surveys 2000 Ottawa, Ontario K1A 0K9: Health Canada 23 68

[B14] Conn JM Annest JL Gilchrist J Sports and recreation related injury episodes in the US population, 1997–99 Inj Prev 2003 9 2 117 123 10.1136/ip.9.2.117 12810736 PMC1730974

[B15] Turner AP Barlow JH Heathcote-Elliott C Long term health impact of playing professional football in the United Kingdom Br J Sports Med 2000 34 5 332 336 10.1136/bjsm.34.5.332 11049141 PMC1756230

[B16] Roos H Lindberg H Gärdsell P Lohmander LS Wingstrand H The prevalence of gonarthrosis and its relation to meniscectomy in former soccer players Am J Sports Med 1994 22 2 219 222 10.1177/036354659402200211 8198190

[B17] Lindberg H Roos H Gärdsell P Prevalence of coxarthrosis in former soccer players Acta Orthop Scand 1993 64 165 167 10.3109/17453679308994561 8498177

[B18] Williams JM Andersen MB Psychosocial antecedents of sport injury: review and critique of stress and injury model J Appl Sport Psych 1998 10 5 25 10.1080/10413209808406375

[B19] Williams JM Andersen MB Tenenbaum G Psychosocial Antecedents of Sports Injury and Interventions for Risk Reduction Handbook of Sport Psychology 2007 Eklund RC: Wiley 379 403

[B20] Junge A The influence of psychological factors on sports injuries. Review of the literature Am J Sports Med 2000 28 5 Suppl S10 S15 11032102 10.1177/28.suppl_5.s-10

[B21] Petrie T Coping skills, competitive anxiety, and playing status: moderating effects on the life stress injury-relationship J Sport Exerc Psych 1993 15 261 274

[B22] Petrie T Psychological antecedents of athletic injuries: the effects of life stress and social support on female collegiate gymnasts Behav Med 1992 18 127 138 10.1080/08964289.1992.9936963 1421746

[B23] Hanin YL Hanin YL Individual Zones of Optimal Functioning (IZOF) Model: Emotion-Performance Relationships in Sport Emotion in Sports 2000 Champaign, Il: Human Kinetics 65 90

[B24] Hanin YL Spielberger CD Emotion in Sports: An Individualized Approach Encyclopedia of Applied Psychology 2004 Oxford, UK: Elsevier Academic Press 739 750

[B25] Devonport TJ Lane AM Hanin YL Emotional states of athletes prior to performance-induced injury J Sports Sci Med 2005 4 382 394 24501552 PMC3899654

[B26] Galambos SA Terry PC Moyle GM Locke SA Lane AM Psychological predictors of injury among elite athletes Br J Sports Med 2005 39 6 351 354 10.1136/bjsm.2005.018440 15911606 PMC1725228

[B27] Raglin JS Kentta G Echemendia R, Moorman CTI A Psychological Approach to Understanding and Preventing Overtraining Syndrome Handbook of Sports Medicine and Athletic Health. Volume 3 2011 Santa Barbara, CA: Praeger 93 111

[B28] Meeusen R Duclos M Foster C Fry A Gleeson M Nieman D Raglin J Rietjens G Steinacker J Urhausen A Prevention, diagnosis, and treatment of the overtraining syndrome: joint consensus statement of the European College of Sport Science and the American College of Sports Medicine Med Sci Sports Exerc 2013 45 1 186 205 10.1249/MSS.0b013e318279a10a 23247672

[B29] Austin MP Ross M Murray C O’Carroll RE Ebmeier KP Goodwin GM Cognitive function in major depression J Affect Disord 1992 25 1 21 29 10.1016/0165-0327(92)90089-O 1624644

[B30] Raglin JS Psychological factors in sport performance: the Mental Health Model revisited Sports Med 2001 31 12 875 890 10.2165/00007256-200131120-00004 11665914

[B31] Kentta G Hassmen P Raglin JS Mood state monitoring of training and recovery in elite kayakers Eur J Sports Sci 2006 4 245 253

[B32] Johnson U Ivarsson A Psychological predictors of sport injuries among junior soccer players Scand J Med Sci Sports 2011 21 129 136 10.1111/j.1600-0838.2009.01057.x 20136759

[B33] Fawkner HJ McMurrary NE Summers JJ Athletic injury and minor life events: a prospective study J Sci Med Sport 1999 2 2 117 124 10.1016/S1440-2440(99)80191-1 10476975

[B34] Moodie EE Stephens DA Marginal Structural Models: unbiased estimation for longitudinal studies Int J Public Health 2011 56 1 117 119 10.1007/s00038-010-0198-4 20931349

[B35] Mittleman MA Maclure M Robins JM Control sampling strategies for case-crossover studies: an assessment of relative efficiency Am J Epidemiol 1995 142 1 91 98 7785679 10.1093/oxfordjournals.aje.a117550

[B36] Ivarsson A Johnson U Psychological factors as predictors of injuries among senior soccer players. A prospective study J Sports Sci Med 2010 9 347 352 24149706 PMC3761721

[B37] Ivarsson A Johnson U Podlog L Psychological predictors of injury occurrence: A prospective investigation of professional swedish soccer players J Sport Rehab 2013 22 19 26 10.1123/jsr.22.1.19 23404909

[B38] Ivarsson A Johnson U Lindwall M Gustafsson H Altemyr M Psychosocial stress as a predictor of injury in elite junior soccer: a latent growth curve analysis J Sci Med Sport 2013 doi:10.1016/j.jsams.2013.10.242. [Epub ahead of print] 10.1016/j.jsams.2013.10.242 24262336

[B39] Berglund B Safstrom H Psychological monitoring and modulation of training load of world-class canoeists Med Sci Sports Exerc 1994 26 8 1036 1040 7968421

[B40] Raglin JS Hanin YL Hanin YL Competitive Anxiety Emotion in Sports 2000 Champaign, Il: Human Kinetics 93 111

[B41] Raglin JS Morgan WP Development of a scale for use in monitoring training-induced distress in athletes Int J Sports Med 1994 15 2 84 88 10.1055/s-2007-1021025 8157374

[B42] O’Connor PJ Morgan WP Raglin JS Psychobiologic effects of 3 d of increased training in female and male swimmers Med Sci Sports Exerc 1991 23 9 1055 1061 1943626

[B43] Turner PE Raglin JS Variability in precompetition anxiety and performance in college track and field athletes Med Sci Sports Exerc 1996 28 3 378 385 8776227 10.1097/00005768-199603000-00014

[B44] Hootman JM Dick R Agel J Epidemiology of collegiate injuries for 15 sports: summary and recommendations J Athl Train 2007 42 2 311 319 17710181 PMC1941297

[B45] Shrier I Meeuwisse WH Matheson GO Wingfield K Steele RJ Prince F Hanley J Montanaro M Injury patterns and injury rates in the circus arts: an analysis of 5 years of data from Cirque du Soleil Am J Sports Med 2009 37 6 1143 1149 10.1177/0363546508331138 19286913

[B46] Spielberger CD Gorsuch RL Lushene R Vagg PR Jacobs GA State-Trait Anxiety Inventory for Adults 1983 Palo Alto, CA: Consulting Psychologists Press Inc.

[B47] Folkman S Lazarus RS Dunkel-Schetter C DeLongis A Gruen RJ Dynamics of a stressful encounter: cognitive appraisal, coping, and encounter outcomes J Pers Soc Psychol 1986 50 5 992 1003 3712234 10.1037//0022-3514.50.5.992

[B48] McNair DM Lorr M Dropplemann LF Profile of Mood States Manual 1992 San Diego, CA: Educational and Testing Service

[B49] Morgan WP Costill DL Flynn MG Raglin JS O’Connor PJ Mood disturbance following increased training in swimmers Med Sci Sports Exerc 1988 20 4 408 414 10.1249/00005768-198808000-00014 3173050

[B50] Spielberger CD Gorsuch RL Lushene PE Vagg PR Jacobs GA Manual for the State-Trait Anxiety Inventory (Form Y) 1983 Palo Alto, CA: Consulting Psychologist Press

[B51] Davey HM Barratt AL Butow PN Deeks JJ A one-item question with a Likert or Visual Analog Scale adequately measured current anxiety J Clin Epidemiol 2007 60 4 356 360 10.1016/j.jclinepi.2006.07.015 17346609

[B52] Lundqvist C Kentta G Raglin JS Directional anxiety responses in elite and subelite young athletes: intensity of anxiety symptoms matters Scand J Exerc Sci Sports 2011 21 6 853 862 10.1111/j.1600-0838.2010.01102.x 22126716

[B53] Lee C Bobko P Self-efficacy beliefs: comparison of five measures J Appl Psychol 1994 79 364 369

[B54] van Hooff ML Geurts SA Kompier MA Taris TW “How fatigued do you currently feel?” Convergent and discriminant validity of a single-item fatigue measure J Occup Health 2007 49 3 224 234 10.1539/joh.49.224 17575403

[B55] Raglin JS Koceja DM Stager JM Harms CA Mood, neuromuscular function, and performance during training in female swimmers Med Sci Sports Exerc 1996 28 3 372 377 8776226 10.1097/00005768-199603000-00013

[B56] Fuller CW Molloy MG Bagate C Bahr R Brooks JHM Donson H Kemp SPT McCrory P McIntosh AS Meeuwisse WH Quarrie KL Raftery M Wiley P Consensus statement on injury definitions and data collection procedures for studies of injuries in rugby union Clin J Sport Med 2007 17 177 181 10.1097/JSM.0b013e31803220b3 17513907

[B57] Dick R Putukian M Agel J Evans TA Marshall SW Descriptive epidemiology of collegiate women’s soccer injuries: National Collegiate Athletic Association Injury Surveillance System, 1988–1989 through 2002–2003 J Athl Train 2007 42 2 278 285 17710177 PMC1941298

[B58] Emery CA Meeuwisse WH Hartmann SE Evaluation of risk factors for injury in adolescent soccer: implementation and validation of an injury surveillance system Am J Sports Med 2005 33 12 1882 1891 10.1177/0363546505279576 16157843

[B59] Core Team R R: A language and environment for statistical computing v. 2.7.1 Vienna, Austria: R Foundation for Statistical Computing; 2007

[B60] Bates D Maechler M Bolker B lme4: Linear Mixed-Effects Models Using S4 Classes R: A Language and Environment for Statistical Computing R Foundation for Statistical Computing 2011 0999375-39 Vienna, Austria

[B61] Yan J Højsgaard S Halekoh U Geepack: Generalized Estimating Equation Package R: A language and environment for statistical computing R Foundation for Statistical Computing 2011 10-18 Vienna, Austria

[B62] Dupont WD Plummer WD PS power and sample size program available for free on the Internet Control Clin Trials 1998 19 589 601 10.1016/S0197-2456(98)00037-3 9875838

[B63] Macaulay AC Commanda LE Freeman WL Gibson N McCabe ML Robbins CM Twohig PL Participatory research maximises community and lay involvement. North American Primary Care Research Group BMJ 1999 319 7212 774 778 10.1136/bmj.319.7212.774 10488012 PMC1116604

[B64] Feltz DL Lirgg CD Singer RN, Hausenblas HA, Janelle CM Self-Efficacy Beliefs of Athletes, Teams and Coaches Handbook of Sport Psychology 2001 2 New York, NY: John Wiley & Sons 340 361

[B65] Shrier I Halle M Psychological predictors of injuries in circus artists: an exploratory study Br J Sports Med 2011 45 433 436 10.1136/bjsm.2009.067751 21047839

[B66] Verhagen E Clarsen B Bahr R A peek into the future of sports medicine: the digital revolution has entered our pitch Br J Sports Med 2014 48 739 740 10.1136/bjsports-2013-093103 24273310

[B67] Kumar S Nilsen WJ Abernethy A Atienza A Patrick K Pavel M Riley WT Shar A Spring B Spruijt-Metz D Hedeker D Honavar V Kravitz R Lefebvre RC Mohr DC Murphy SA Quinn C Shusterman V Swendeman D Mobile health technology evaluation: the mHealth evidence workshop Am J Prev Med 2013 45 2 228 236 10.1016/j.amepre.2013.03.017 23867031 PMC3803146

[B68] Ekegren CL Gabbe BJ Finch CF Injury reporting via SMS text messaging in community sport Inj Prev 2014 doi: 10.1136/injuryprev-2013-041028. [Epub ahead of print] 10.1136/injuryprev-2013-041028 24413851

