# Supplementary material for: Procedures for assessing psychological predictors of injuries in circus artists: a pilot prospective study
Source: BMC Med Res Methodol. 2014 Jun 11;14:77. doi: 10.1186/1471-2288-14-77 (PMC4064279; doi:10.1186/1471-2288-14-77)
Supplement: Additional file 1 — Daily questionnaire. [file 1471-2288-14-77-S1.doc]

## *Additional file 1: Daily Questionnaire*

1. How do you feel today (circle your response):

| Very very good | Very good | Good | Average | Bad | Very bad | Very very bad |
| --- | --- | --- | --- | --- | --- | --- |

2. How many hours did you sleep last night? _____.

Did you sleep soundly? Yes No

3. Have you felt sick over the past day? Yes No

If you said yes, was it something you ate or drank? Yes No

| 4. How do your muscles feel? (Fill in each blank with a number) | (1) Very very good |
| --- | --- |
| Whole Body: _______ | (2) Very good |
| Arms: ________ | (3) Good |
| Legs: ________ | (4) Tender but not sore |
|  | (5) Sore |
|  | (6) Very sore |
|  | (7) Very, very sore |

5. How do you feel about your own UPCOMING performance(s) today (circle your response)?

| Totally confident (comfortable) | Very confident | Somewhat confident | Neither confident nor unconfident | Somewhat unconfident | Very unconfident | Totally unconfident |
| --- | --- | --- | --- | --- | --- | --- |

6. How nervous are you about today’s UPCOMING performance(s) compared to before the optimal performances you have had in your CURRENT ROLE (circle your response)?

| A lot more nervous | Somewhat more nervous | Neither more or less nervous | Somewhat less nervous | A lot less nervous |
| --- | --- | --- | --- | --- |

7. How fatigued do you currently feel (circle your response)?

| 1 | 2 | 3 | 4 | 5 | 6 | 7 | 8 | 9 | 10 |
| --- | --- | --- | --- | --- | --- | --- | --- | --- | --- |
| Not at all |  |  |  |  |  |  |  |  | Extremely |
